# Anti-HmuY Antibodies Specifically Recognize *Porphyromonas gingivalis* HmuY Protein but Not Homologous Proteins in Other Periodontopathogens

**DOI:** 10.1371/journal.pone.0117508

**Published:** 2015-02-06

**Authors:** Michał Śmiga, Marcin Bielecki, Mariusz Olczak, John W. Smalley, Teresa Olczak

**Affiliations:** 1 Laboratory of Biochemistry, Faculty of Biotechnology, University of Wroclaw, 14A F. Joliot-Curie St., 50-383 Wroclaw, Poland; 2 School of Dentistry, University of Liverpool, Research Wing, Daulby Street, Liverpool L69 3GN, United Kingdom; Public Health Research Institute at RBHS, UNITED STATES

## Abstract

Given the emerging evidence of an association between periodontal infections and systemic conditions, the search for specific methods to detect the presence of *P. gingivalis*, a principal etiologic agent in chronic periodontitis, is of high importance. The aim of this study was to characterize antibodies raised against purified *P. gingivalis* HmuY protein and selected epitopes of the HmuY molecule. Since other periodontopathogens produce homologs of HmuY, we also aimed to characterize responses of antibodies raised against the HmuY protein or its epitopes to the closest homologous proteins from *Prevotella intermedia* and *Tannerella forsythia*. Rabbits were immunized with purified HmuY protein or three synthetic, KLH-conjugated peptides, derived from the *P. gingivalis* HmuY protein. The reactivity of anti-HmuY antibodies with purified proteins or bacteria was determined using Western blotting and ELISA assay. First, we found homologs of *P. gingivalis* HmuY in *P. intermedia* (PinO and PinA proteins) and *T. forsythia* (Tfo protein) and identified corrected nucleotide and amino acid sequences of Tfo. All proteins were overexpressed in *E. coli* and purified using ion-exchange chromatography, hydrophobic chromatography and gel filtration. We demonstrated that antibodies raised against *P. gingivalis* HmuY are highly specific to purified HmuY protein and HmuY attached to *P. gingivalis* cells. No reactivity between *P. intermedia* and *T. forsythia* or between purified HmuY homologs from these bacteria and anti-HmuY antibodies was detected. The results obtained in this study demonstrate that *P. gingivalis* HmuY protein may serve as an antigen for specific determination of serum antibodies raised against this bacterium.

## Introduction

Periodontitis is a group of multifactorial, inflammatory infectious diseases, initiated by an ecological shift in the composition of subgingival biofilm, resulting in inflammation and destruction of tooth-supporting tissues, eventually leading to tooth loss [1–3]. From a clinical point of view, chronic periodontitis is characterized by deep periodontal pockets, resulting from the loss of alveolar bone and connective tissue attachment to the tooth. The severity of bleeding upon probing depends on the intensity of the gingival inflammation. Most of the tissue damage results from both direct destructive effects of the pathogenic plaque microorganisms themselves and from the exaggerated host responses to bacterial challenges.

Several studies have demonstrated that about 700 species are capable of colonizing the adult oral cavity [4,5]. Analysis of bacterial species isolated from subgingival samples has revealed the presence and relative abundance of periodontal pathogens, including the “red complex” members (*Porphyromonas gingivalis*, *Tannerella forsythia* and *Treponema denticola*), associated with the clinical features of chronic periodontitis [4–7]. In addition, *Prevotella intermedia*, a member of the "orange complex", serves as a bridging species through binding to members of the "red complex" [8,9]. Among periodontal pathogens, *P*. *gingivalis* is considered the main etiologic agent and a key pathogen responsible for initiation and progression of chronic periodontitis [10,11].

*P*. *gingivalis* is a heme auxotroph, and therefore the uptake of this compound is essential for bacterial survival and the ability to establish an infection. To acquire iron and heme, *P*. *gingivalis* uses several sophisticated mechanisms [12], and some of them, *e*.*g*., the *hmu* system [13], are well characterized. One of its components, namely HmuY, is a membrane-associated heme-binding lipoprotein [14,15]. Heme uptake served by this hemophore-like protein is a novel system which was identified for the first time in *P*. *gingivalis* [13–21].

*P*. *gingivalis* can enter gingival epithelial and immune cells, remaining viable and capable of spreading among host cells, thus contributing to its survival in the oral cavity [22–25]. The bacterium produces various secreted and structural components that directly cause destruction of periodontal tissues and play a crucial role in the induction of innate immune responses [10]. It has been demonstrated that *P*. *gingivalis* can also spread systemically to other tissues [26–28]. Our data also suggest that the *P*. *gingivalis* HmuY could constitute a mechanism for stimulation of the host immune system and be of particular importance in development of chronic periodontitis [29–32]. *P*. *gingivalis* HmuY is constitutively produced, but at higher levels when bacteria grow under low-iron/heme conditions or are a biofilm constituent [14]. Importantly, the protein may be released from the bacterium in the form of outer-membrane vesicles [14,33] or may be shed from the membrane surface in the soluble form [15]. Therefore, HmuY production and its release to the surrounding environment could be of significance in periodontal pockets, where the biofilm provides persistent bacterial colonization. After entering the periodontal pocket epithelium, free-soluble bacterial products may readily gain access to the blood vascular network and spread systemically. Indeed, we have demonstrated that patients with chronic periodontitis produce higher levels of anti-HmuY antibodies compared to healthy subjects [29] and patients with gingivitis or aggressive periodontitis (S.C. Trindade, T. Olczak, unpublished data).

Given the emerging evidence of an association between periodontal infections and systemic conditions such as diabetes mellitus, rheumatoid arthritis, cardiovascular and respiratory diseases [28,34,35], as well as increasing resistance of bacteria against antibiotics, the search for methods for the specific detection of *P*. *gingivalis* and its inactivation is highly important. Previously, we developed a simple but efficient assay for specific and sensitive detection of *P*. *gingivalis* using the *hmuY* gene sequence and qPCR [36]. Based on our results it has emerged that the unique *hmuY* sequence may serve as one of molecular markers of *P*. *gingivalis*. The aim of this study was to characterize antibodies raised against purified *P*. *gingivalis* HmuY protein and selected epitopes of the HmuY molecule. Since other periodontopathogens produce homologs of HmuY, we also aimed to characterize responses of antibodies raised against the HmuY protein or its epitopes to the closest homologous proteins from *P*. *intermedia* and *T*. *forsythia*.

## Materials and Methods

### Ethics statement

All animal protocols and procedures used to produce custom designed polyclonal antibodies purchased from GenScript Corporation (GenScript USA Inc., Piscataway, NJ 08854) were approved by the GenScript Corporation Institutional Animal Care and Use Committee (IACUC, #ANT11-001). The Office of Laboratory Animal Welfare (OLAW) of the U.S. National Institutes of Health (NIH) provides guidance and interpretation of the Public Health Service (PHS) Policy on Humane Care and Use of Laboratory Animals. GenScript received OLAW's Animal Welfare Assurance. OLAW's Animal Welfare Assurance accentuates the responsibilities and procedures of GenScript regarding the care and use of laboratory animals. The GenScript developed partnership with the Association for Assessment and Accreditation of Laboratory Animal Care (AAALAC) International, which is a private, nonprofit organization that promotes the humane treatment of animals in science through voluntary accreditation and assessment programs. GenScript has earned AAALAC accreditation, demonstrating their commitment to responsible animal care and use through ongoing voluntary participation in AAALAC programs.

### Bacterial strains and growth conditions

*P*. *gingivalis* A7436 and ATCC 33277, *P*. *intermedia* 17, and *T*. *forsythia* ATCC 43037 were grown under anaerobic conditions as described previously [36,37].

### Construction of expression plasmids

To construct expression plasmids containing sequences encoding *P*. *gingivalis* HmuY homologs from *P*. *intermedia* and *T*. *forsythia*, DNA sequences encoding predicted mature proteins were PCR-amplified using the genomic DNAs as templates and primers listed in Table 1. For this purpose, *P*. *intermedia* DNA sequences encoding PinA (NCBI accession number WP_014709321) and PinO (NCBI accession number WP_014708291) were used. In the case of DNA encoding *T*. *forsythia* Tfo, some discrepancies in the gene sequence compared to the DNA sequence deposited in databases (NCBI accession number YP_005014932) were found. Therefore, in this study we determined the corrected DNA sequence encoding Tfo. All amplified PCR products were ligated into the pTriEx-4 vector (Merck), resulting in sequences encoding untagged proteins.

**Table 1:** Primers designed and used in this study.

Primer	5’→3’ sequence (restriction sites are underlined)	Amplification product length (bp)	Locus ID	Description
FPin0009_Esp3I BsmBI	atctcgtctcgcatgagcaaggacaacaacgacgac	645	PIN_0009	Amplifies sequence coding for untagged *P*. *intermedia* HmuY homolog (PinO) without signal peptide sequence and cysteine residue (20–225 aa)
RPin0009_STOP XhoI	gatctcgagttacttcgctttctttatgaacttatag			
FPIN_A0726 BsaI	acgtggtctcgcatgagcaatgatgacccaactccaaaac	705	PIN_A0726	Amplifies sequence coding for untagged *P*. *intermedia* HmuY homolog (PinA) without signal peptide sequence and cysteine residue (21–246 aa)
RPIN_A0726_STOP XhoI	cgtctcgagttaattcttcttgacaaatttatacttgaagc			
FHmuY_Tf_NcoI	cgtccatggacaagaaagacgacgtaaaag	605	Bfo_2078	Amplifies sequence coding for untagged *T*. *forsythia* HmuY homolog (Tfo) without signal peptide sequence and cysteine residue (22–216 aa)
RHmuY_Tf_STOP XhoI	agtctcgagttatttcggttgaaattcgtaattaaaag			

### Overexpression and purification of proteins

*P*. *gingivalis* HmuY lacking the first 25 amino acid residues (NCBI accession number CAM 31898) was overexpressed using a pHmuY11 plasmid and *Escherichia coli* ER2566 cells (New England Biolabs) and purified from a soluble fraction of the *E*. *coli* cell lysate as described previously [13]. As the soluble protein shed from the outer membrane, the purified HmuY lacked the signal peptide and first five amino acid residues (CGKKK) of the nascent secreted protein [14,15]. *P*. *intermedia* PinA, PinO and *T*. *forsythia* Tfo were overexpressed in *E*. *coli* ER2566 and Rosetta (DE3) strains (New England Biolabs or Merck), respectively. PinA, PinO and Tfo, lacking predicted signal peptides (Table 1), were prepared using purification procedures established in our laboratory. All proteins were purified from whole *E*. *coli* cell lysates obtained after sonication and centrifugation, as described for *P*. *gingivalis* HmuY [13] with some modifications. Briefly, PinO, PinA and Tfo purification was carried out using DEAE-Sephacel chromatography (50 mM Tris/HCl buffer, pH 7.6, containing 30 mM NaCl). Then, hydrophobic chromatography using *Phenyl* Superose (HR 5/5, Pharmacia) was used. The proteins were bound to the chromatographic resin in 50 mM sodium phosphate buffer, pH 7.0, containing 2 M ammonium sulfate, and eluted using linear salt gradient 2.0–0.0 M. As the final step, gel filtration using Sephadex G-75 (50 mM Tris/HCl buffer, pH 7.6, containing 400 mM NaCl) was used.

### Immunization of rabbits

Custom designed polyclonal antibodies were purchased from the commercial company (GenScript USA Inc.), which is a dedicated biology research service provider. *P*. *gingivalis* HmuY and three synthetic peptides derived from the amino acid sequence of HmuY, conjugated with keyhole limpet hemocyanin (KLH), were used to immunize rabbits. Each experimental animal had one ear tag with lot number and one animal was kept in one cage with cage card. Animals were maintained at 16–22°C and relative humidity 30–70%. Polyclonal antibodies were produced by subcutaneously injecting New Zealand white rabbits at weight 2.0–2.5 kg (2 rabbits per each antigen, 8 rabbits during the entire experiment). In addition, sera from 2 rabbits immunized with the purified HmuY protein in our previous study [14] were used. The rabbits received one injection with the antigen (0.2 mg per injection) emulsified 1:1 (0.25 ml per rabbit) with Freund’s complete adjuvant, and the same amount of antigen with Freund’s incomplete adjuvant on day14, 35 and 56. Before immunization and after immunization on day 21, 42, and 63 blood was collected (2.5 ml per animal) from ear margin veins. All animals were healthy trough the entire experiment and all serum samples were properly collected. Pre-immune sera (protocol #SC1088) and immune sera after test and final bleeds (protocols #SC1247 and # SC3031), containing anti-HmuY1 (purified protein), anti-HmuY1–1 (GKKKDEPNQPSTPEC), anti-HmuY1–2 (SKGEVVNVTDYKNDC), and anti-HmuY1–3 (CEMGPDGHQMEYEEQG) antibodies were used for all experiments.

### Sodium dodecyl sulfate polyacrylamide gel electrophoresis (SDS-PAGE) and Western blotting

The reactivity of respective antibodies was investigated using purified proteins or bacterial cell lysates. Bacterial cultures were centrifuged for 20 min at 20,000×*g* at 4°C. Bacterial pellets were washed with 20 mM sodium phosphate buffer, pH 7.4, containing 140 mM NaCl (PBS) and suspended in PBS to OD_600_ = 1.0. Samples corresponding to 20 μl of the bacterial culture or protein samples (1, 10 and 100 ng or 1 and 5 μg per lane) were denatured and separated on 12% SDS-PAGE gels, and subsequently transferred onto nitrocellulose membranes (Whatman). Nonspecific binding sites were blocked with 5% skim milk in PBS with addition of 0.1% Tween (PBST). HmuY or its epitopes were visualized with respective sera and secondary goat anti-rabbit IgG antibodies conjugated with horseradish peroxidase (HRP, Sigma). All sera were used at 1:10,000 dilution. The reaction was developed using chemiluminescence reagents (Western Lightning *Plus*-ECL, Perkin Elmer).

### Enzyme-linked immunosorbent assay (ELISA)

96-well polystyrene plates (Polysorp, Nunc) were coated for 1 h at 37°C with respective proteins (1, 2, 5, 10, 25, 50, 75 and 100 ng per well) or live bacterial cells (100 μl; OD_600_ = 1.0) prepared in PBS. The plates were washed three times with 200 μl of PBS prior to blocking overnight at 4°C with 200 μl of 2% bovine serum albumin (BSA) dissolved in PBS and then washed five times with 200 μl of PBS. Diluted sera were prepared in PBS and incubated for 1 h at 37°C. After washing, antibody binding was detected using goat anti-rabbit IgG conjugated with HRP at 1:10,000 dilution. After five final washes, a substrate solution (100 μl) containing 0.05% *o*-phenylenediamine (Sigma) with 0.01% H_2_O_2_ was added for color development at room temperature. The reaction was stopped after 15 min by adding 25 μl of 12.5% H_2_SO_4_ and the absorbance was measured at 450 nm using a Multiskan Ascent microplate reader (Thermo Electron Corporation).

### Construction of three-dimensional (3D) protein structure of HmuY homologs

PinA, PinO and Tfo protein structures were modeled using the Phyre2 modeling server (http://www.sbg.bio.ic.ac.uk/phyre2) and appropriate templates (PinA—PDB ID: 3U22, crystal structure of a putative HmuY-like heme-binding protein BVU_2192 from *Bacteroides vulgatus* ATCC 8482; PinO—PDB IDs: 3U22, 3H8T, crystal structure of *P*. *gingivalis* heme-binding protein HmuY and PDB ID: 4GBS, crystal structure of a putative lipoprotein BF2707 from *Bacteroides fragilis* NCTC 9343; Tfo—PDB IDs: 3U22, 3H8T, 4GBS). The resulting protein models were refined using ModRefiner (http://zhanglab.med.umich.edu/ModRefiner/) and evaluated using the PSVS online server (PROCHECK, Verify3, MolProbity, ProSA; http://psvs-15-dev.nesg.org/). The best models were selected on the basis of overall G-factor and Ramachandran plot scores and further analyzed using Verify3D (http://nihserver.mbi.ucla.edu/Verify_3D/) and ERRAT (http://nihserver.mbi.ucla.edu/ERRATv2/). Protein models were visualized using PyMOL software (The Open-Source PyMOL Molecular Graphics System Version 0.99r6 Schrödinger, LLC, New York, NY, USA).

## Results and Discussion

Infection with periodontopathogens leads to humoral immune responses with elevated serum antibodies to periodontal species in patients with periodontitis [29,38–44]. In addition to *P*. *gingivalis*, antibody responses to several other periodontopathogens have been found, including *P*. *intermedia* [45,46] and *T*. *forsythia* [47]. More importantly, recent data demonstrated that an elevated antibody level against *P*. *gingivalis* indicated advanced periodontal disease and suggested progression of atherosclerosis, hypertension and rheumatoid arthritis [48–52]. Therefore, efficient screening of patients is essential for estimation of general health and disease stage as well as subsequent treatment efficiency. In our recent study, we presented an extensive phylogenetic analysis of *P*. *gingivalis* HmuY protein and the *hmuY* gene [36]. The HmuY from *P*. *gingivalis* was placed at the base of other Bacteroidia sequences represented by *Bacteroides*, *Prevotella* and *Tannerella*. In contrast, low identity to homologous sequences found in other bacteria was observed. We also demonstrated the presence of antibodies directed against *P*. *gingivalis* HmuY in sera of patients with chronic periodontitis [29].

**Fig 1:**
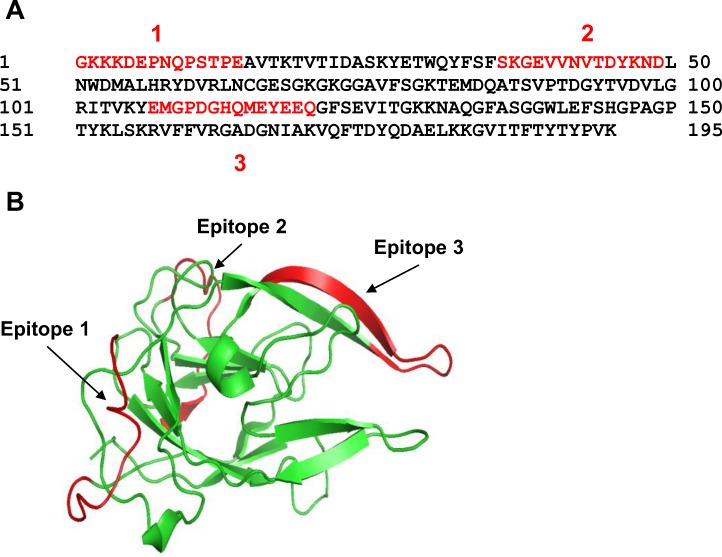
Presentation of *P*. *gingivalis* HmuY epitopes in amino acid sequence (A) and three-dimensional protein structure (B). Epitopes analyzed are indicated in red.

**Fig 2:**
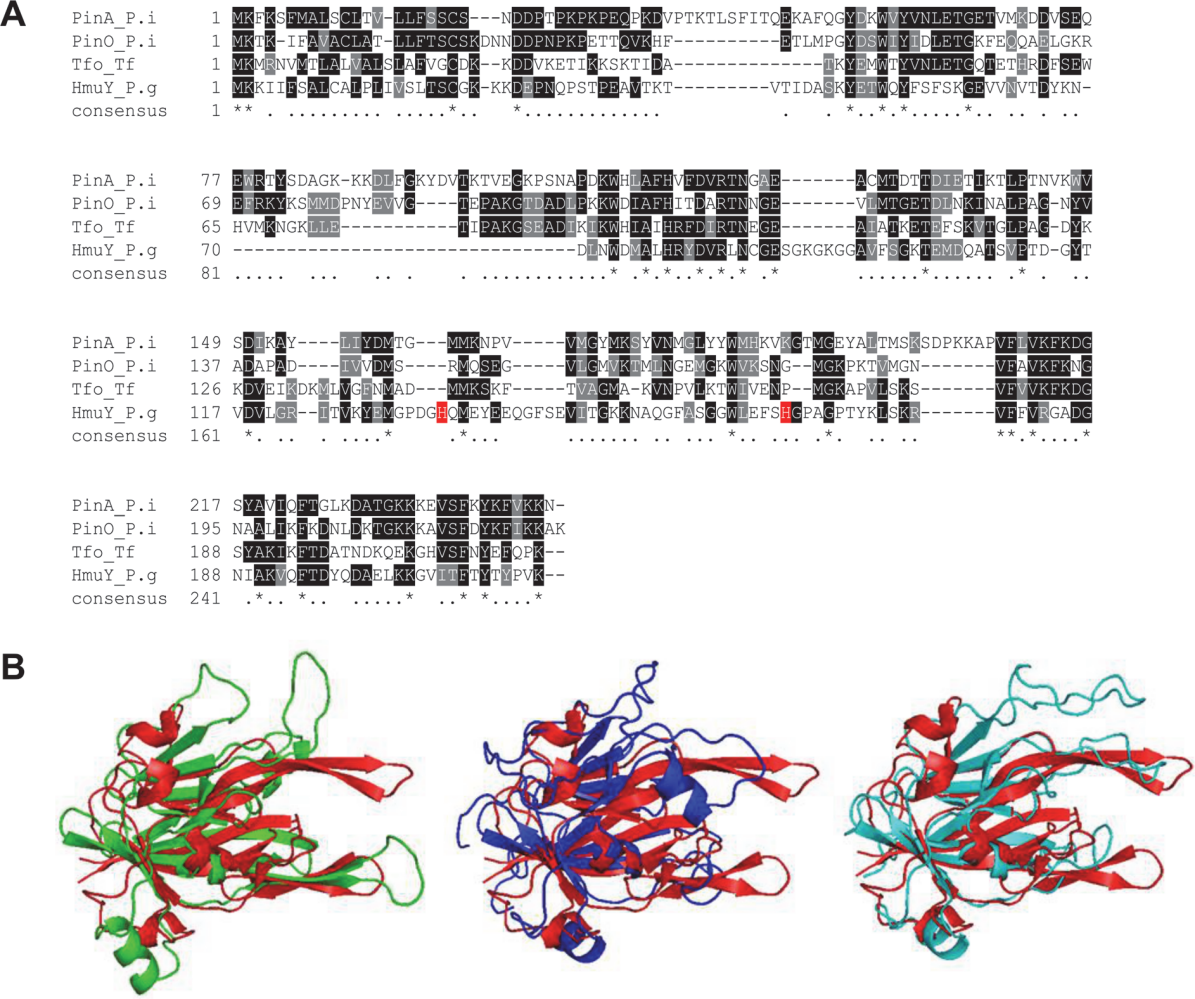
Comparison of *P*. *gingivalis* HmuY and its homologs from *P*. *intermedia* and *T*. *forsythia*. Amino acid alignment (A) and approximated protein structures (B). *P*. *intermedia* PinA is shown in green, *P*. *intermedia* PinO in navy blue, *T*. *forsythia* Tfo in light blue, and *P*. *gingivalis* HmuY (PDB ID: 3H8T) in red. PinA, PinO and Tfo structures were modeled using Phyre2 modeling server and appropriate templates (PinA—PDB IDs: 3U22; PinO—PDB IDs: 3U22, 3H8T, 4GBS; Tfo—PDB IDs: 3U22, 3H8T, 4GBS).

**Fig 3:**
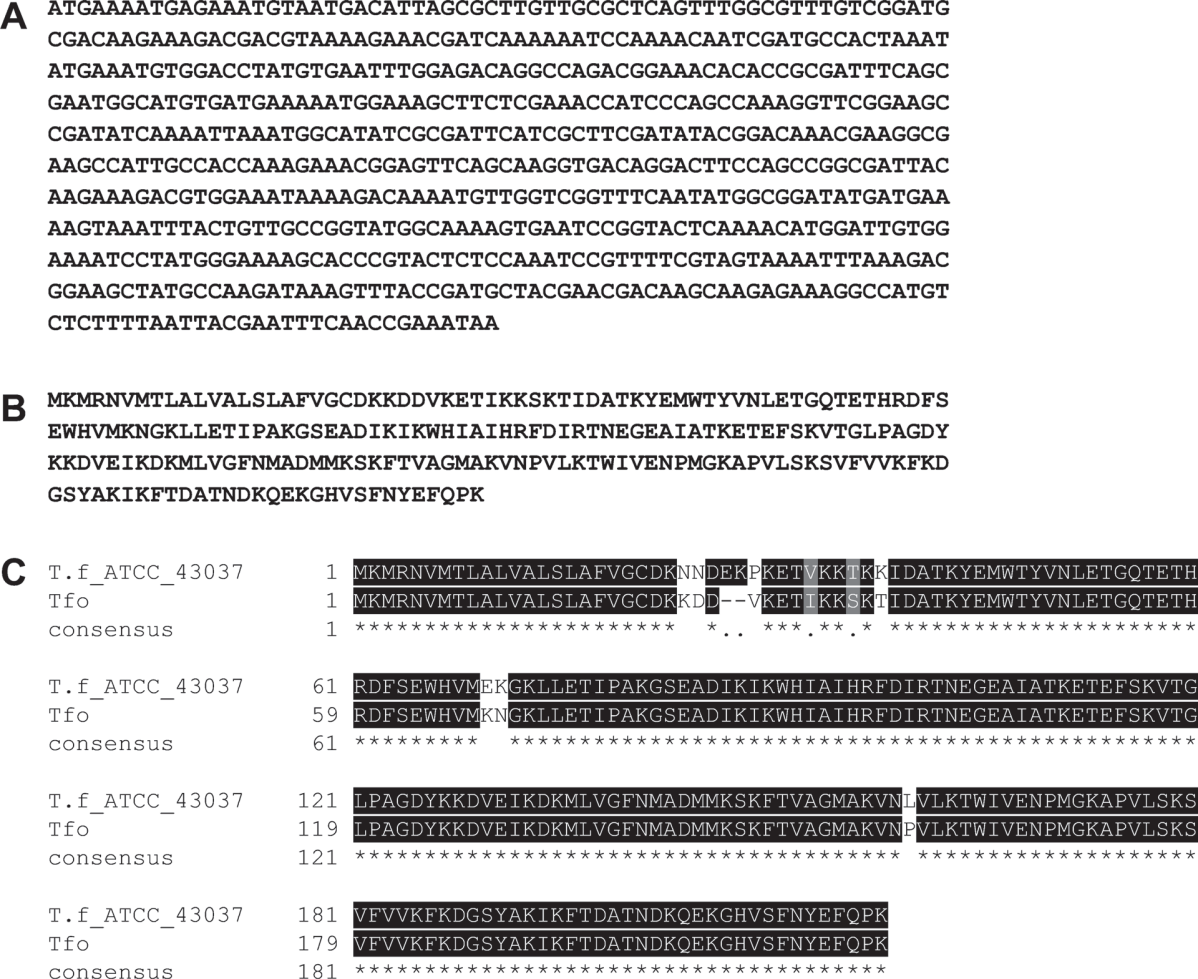
Identification of DNA (A) and amino acid (B) sequences of *T*. *forsythia* Tfo protein. Differences between the amino acid sequence deposited in the database (NCBI ID: YP_005014932) and the sequence identified in this study (EMBL accession number LN624459) are also shown (C).

This study extends our previously published data and presents characterization of the HmuY protein with regard to its future application to determine specific anti-*P*. *gingivalis* antibodies in serum. The best antigen targets are bacterial components, which are constitutively expressed and secreted from the bacterium or exposed on the pathogen’s surface. The use of whole-sonicate antigens, which encompass many of the *P*. *gingivalis* proteins [53,54], would increase the possibility of detecting responses, but also increase the potential for false positive results because of cross-reacting antibodies to components of other bacteria. Therefore, to detect specific anti-*P*. *gingivalis* antibodies which do not cross react with components produced by other bacteria, we employed purified HmuY protein or its selected epitopes as antigens (Fig. 1). Since other periodontopathogens produce HmuY homologs, we aimed to characterize responses of antibodies raised against HmuY protein or its epitopes to the most homologous proteins from *P*. *intermedia* and *T*. *forsythia*. Taking into account the amino acid sequences and the theoretically modeled three-dimensional protein structures, we found the two closest HmuY homologs in *P*. *intermedia* 17, termed here PinA and PinO, and one HmuY homolog from *T*. *forsythia* ATCC 43037, termed here Tfo (Fig. 2). For the latter, we identified corrected DNA (Fig. 3A) and amino acid (Fig. 3B) sequences (EMBL accession number LN624459). It appears that previously in databases a nucleotide sequence possessing a 6-bp insertion and nucleotide substitutions has been deposited. In contrast, the amino acid sequence of Tfo identified in our laboratory contains both a two-amino-acid deletion and substitutions of nine amino acids (Fig. 3C). We did not identify the HmuY homolog in *T*. *denticola*, the third member of the "red complex" of periodontopathogens. In this study we overexpressed all proteins and established purification procedures for the PinA, PinO and Tfo proteins.

**Fig 4:**
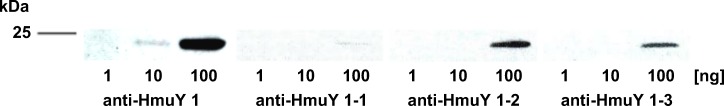
Reactivity of anti-HmuY antibodies with purified *P*. *gingivalis* HmuY protein. Antibodies raised against purified HmuY protein (anti-HmuY 1) or epitope 1 (anti-HmuY 1–1), epitope 2 (anti-HmuY 1–2), and epitope 3 (anti-HmuY 1–3) were analyzed using Western blotting. In Figs 4–9, representative data for one immune serum for each antigen are shown out of 4 immune sera containing antibodies raised against HmuY protein and out of 2 immune sera containing antibodies raised against each epitope.

**Fig 5:**
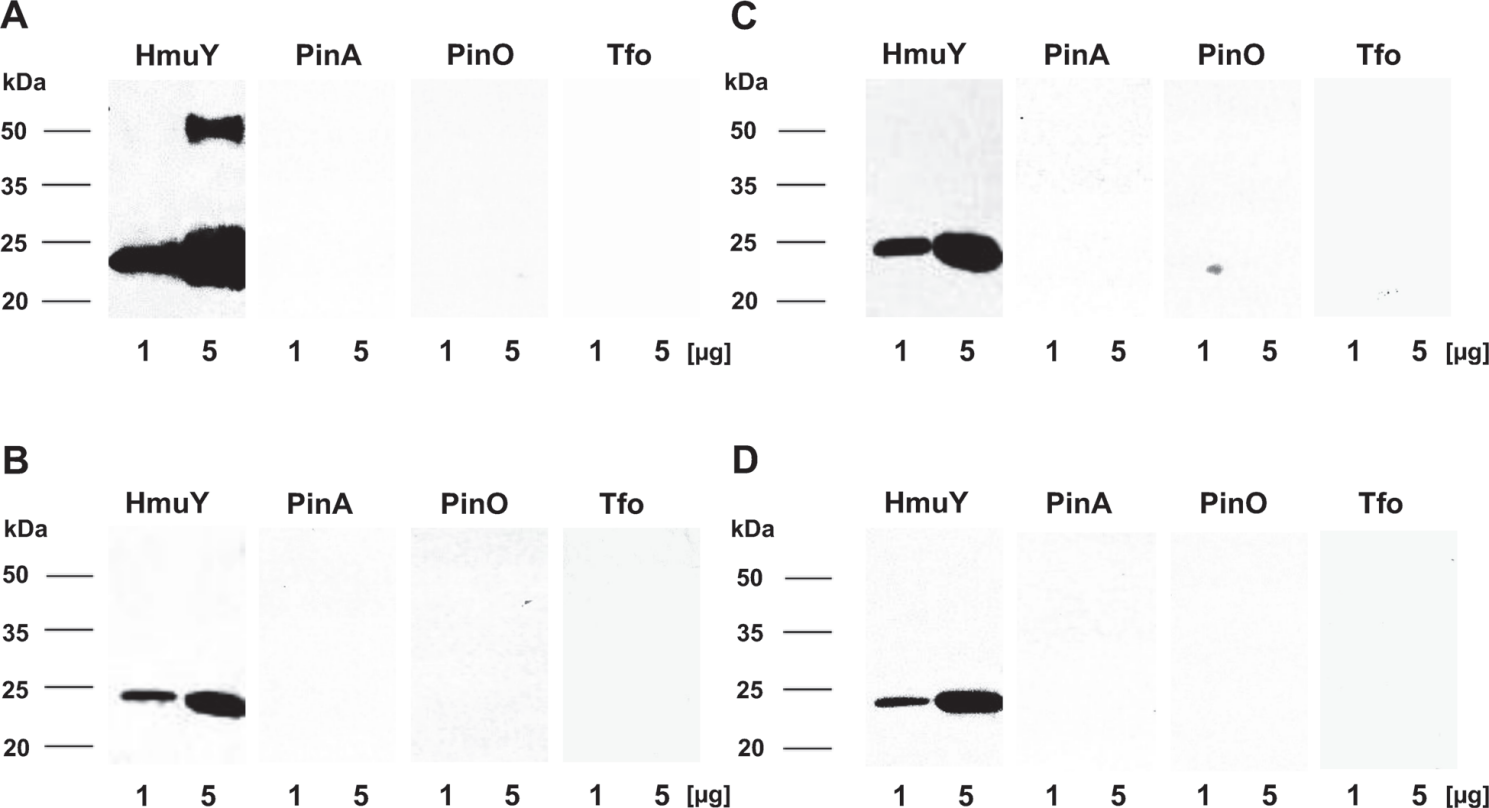
Reactivity of anti-HmuY antibodies with purified *P*. *gingivalis* HmuY or its homologs from *P*. *intermedia* and *T*. *forsythia*. *P*. *intermedia* PinA, PinO and *T*. *forsythia* Tfo were overexpressed, purified and analyzed using Western blotting and antibodies raised against purified HmuY protein (anti-HmuY 1) (A), epitope 1 (anti-HmuY 1–1) (B), epitope 2 (anti-HmuY 1–2) (C), and epitope 3 (anti-HmuY 1–3) (D).

**Fig 6:**
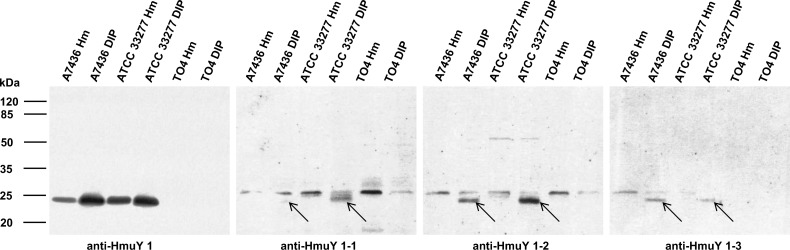
Reactivity of anti-HmuY antibodies with *P*. *gingivalis* wild-type (A7436 and ATCC 33277) and *hmuY* mutant (TO4) strains. Bacteria were cultured in high iron/heme (Hm) or low-iron/heme (DIP) media. Western blotting was carried out using antibodies raised against purified HmuY protein (anti-HmuY 1), epitope 1 (anti-HmuY 1–1), epitope 2 (anti-HmuY 1–2), and epitope 3 (anti-HmuY 1–3). Arrows denote location of bands corresponding to *P*. *gingivalis* HmuY protein.

First we characterized all antibodies produced in rabbits against purified HmuY protein using Western blotting. For this purpose, we examined antibodies against the purified HmuY protein which were raised in 4 rabbits, and antibodies against each synthetic peptide which were raised in 3 pairs of rabbits. We did not observe any significant differences in reactivity between antigens tested and the sera of the different animals (data not shown). Therefore, in this study representative results from single rabbits only are shown. As shown in Fig. 4, the denatured HmuY protein reacted with antibodies directed against the HmuY protein as well as with antibodies directed against all three epitopes, with the highest affinity towards the purified protein and epitope 2. Further analyses showed that antibodies raised against the purified HmuY protein did not recognize HmuY homologs from *P*. *intermedia* and *T*. *forsythia* (Fig. 5A). This strongly demonstrates that antibodies raised against *P*. *gingivalis* HmuY recognize only this antigen, but not homologous proteins from other periodontopathogens. Also, whilst antibodies directed against epitopes 1, 2, and 3 reacted with purified HmuY protein, they did not recognize HmuY homologs examined in this study (Fig. 5B, 5C, 5D). Similar experiments performed with denatured *P*. *gingivalis* cell lysates showed that antibodies raised against purified HmuY protein (anti-HmuY 1) exhibited the highest specificity and recognized the HmuY protein only (Fig. 6). Other antibodies were less specific, and in addition to detection of HmuY protein (Fig. 6, indicated with arrows), they cross reacted with other *P*. *gingivalis* antigens. This non-specific effect was corroborated when the reactivity of pre-immune serum was tested with *P*. *gingivalis* lysates (data not shown). Based on our data we concluded that antibodies raised against HmuY protein (most sensitive and specific) and epitope 2 (less sensitive and specific) detected HmuY protein compared to antibodies raised against epitope 1 and 3. We did also not observe significant differences between two different *P*. *gingivalis* strains used. The *P*. *gingivalis* strain A7436 was originally isolated from a refractory periodontitis patient. This strain is highly similar (both in its genetic background and phenotypic properties) to strain W83. Both strains belong to a group of more virulent *P*. *gingivalis* isolates and are often found in patients with chronic periodontitis [36]. Conversely, *P*. *gingivalis* ATCC 33277 strain was used because it belongs to a less virulent group of strains and is known to exhibit a different genetic background and attenuated virulence properties.

**Fig 7:**
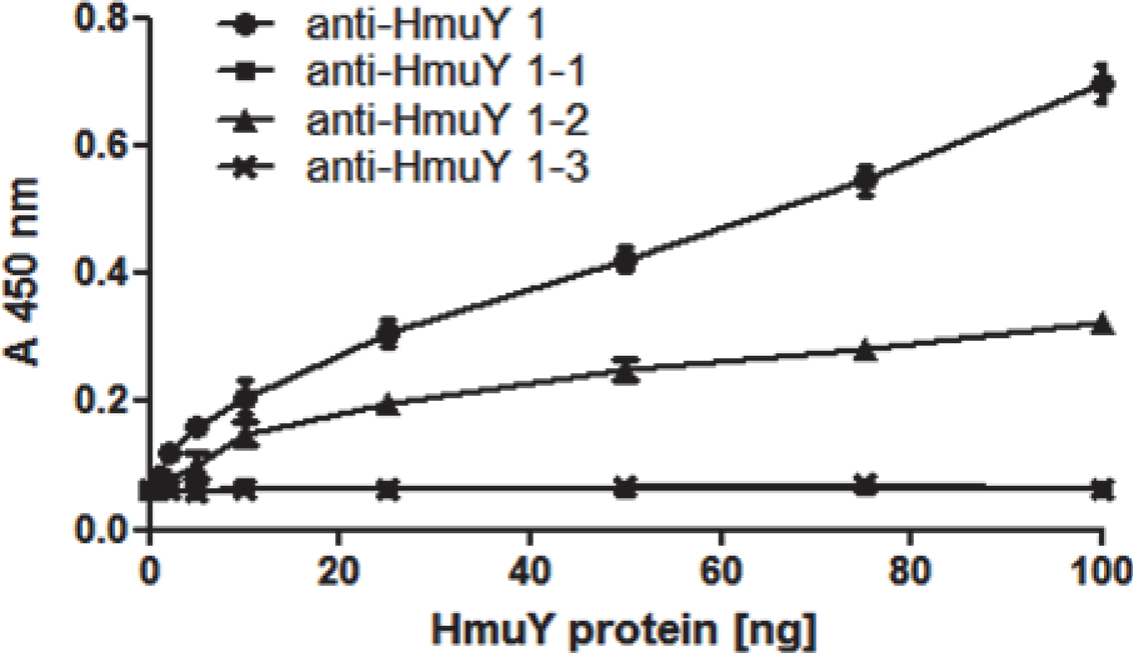
Reactivity of anti-HmuY antibodies with *P*. *gingivalis* HmuY protein. ELISA was carried out using antibodies raised against purified HmuY protein (anti-HmuY 1), epitope 1 (anti-HmuY 1–1), epitope 2 (anti-HmuY 1–2), and epitope 3 (anti-HmuY 1–3). Overexpressed and purified *P*. *gingivalis* HmuY was used as an antigen.

**Fig 8:**
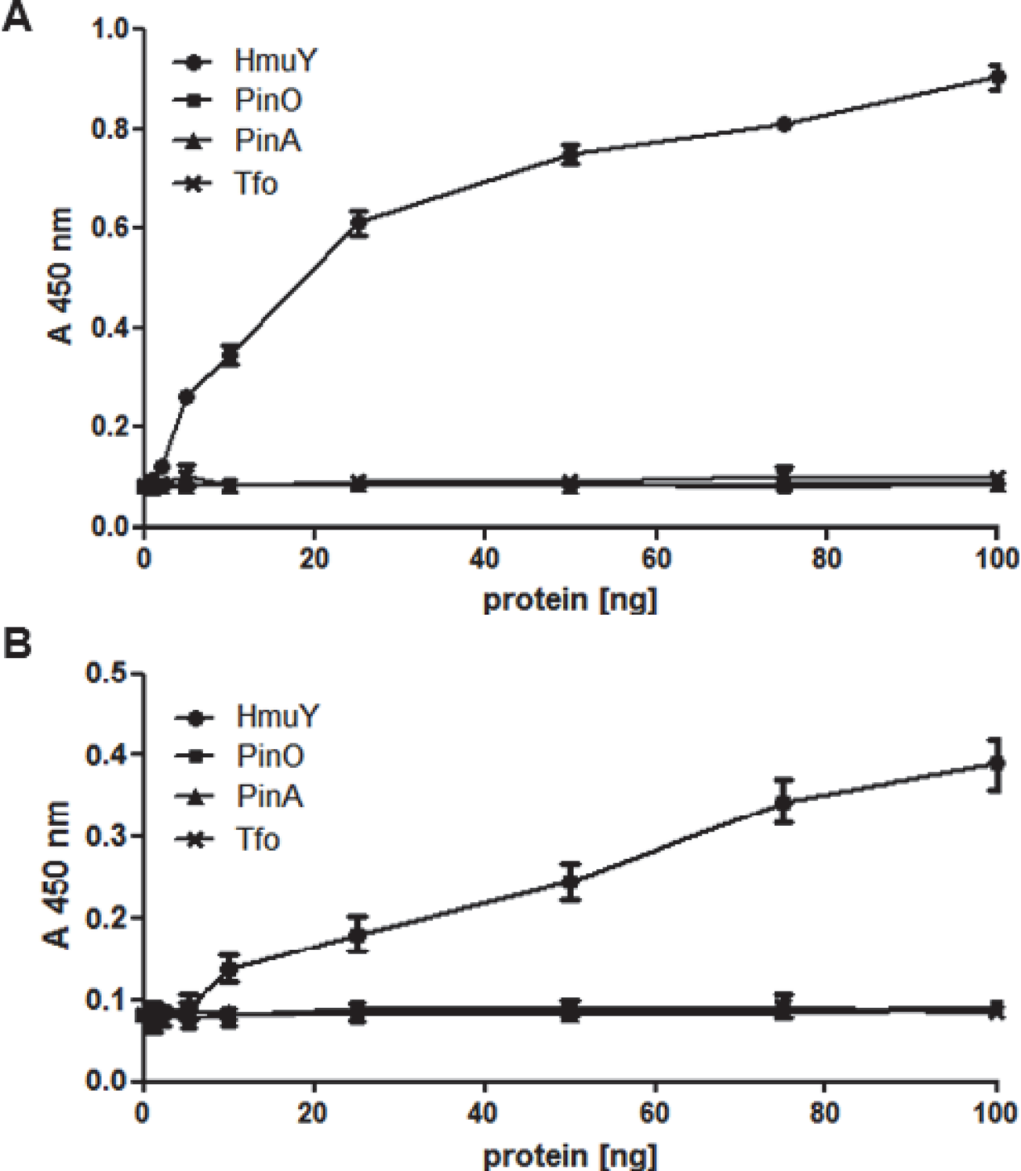
Reactivity of anti-HmuY antibodies with *P*. *gingivalis* HmuY protein or its homologs from *P*. *intermedia* and *T*. *forsythia*. ELISA was carried out using antibodies raised against purified HmuY protein (anti-HmuY 1) (A) and epitope 2 (anti-HmuY 1–2) (B). No reactivity was observed for anti-HmuY 1–1 (epitope 1) and anti-HmuY 1–3 (epitope 3) antibodies. Overexpressed and purified HmuY (*P*. *gingivalis*), PinA, PinO (*P*. *intermedia*) and Tfo (*T*. *forsythia*) proteins were used as antigens.

**Fig 9:**
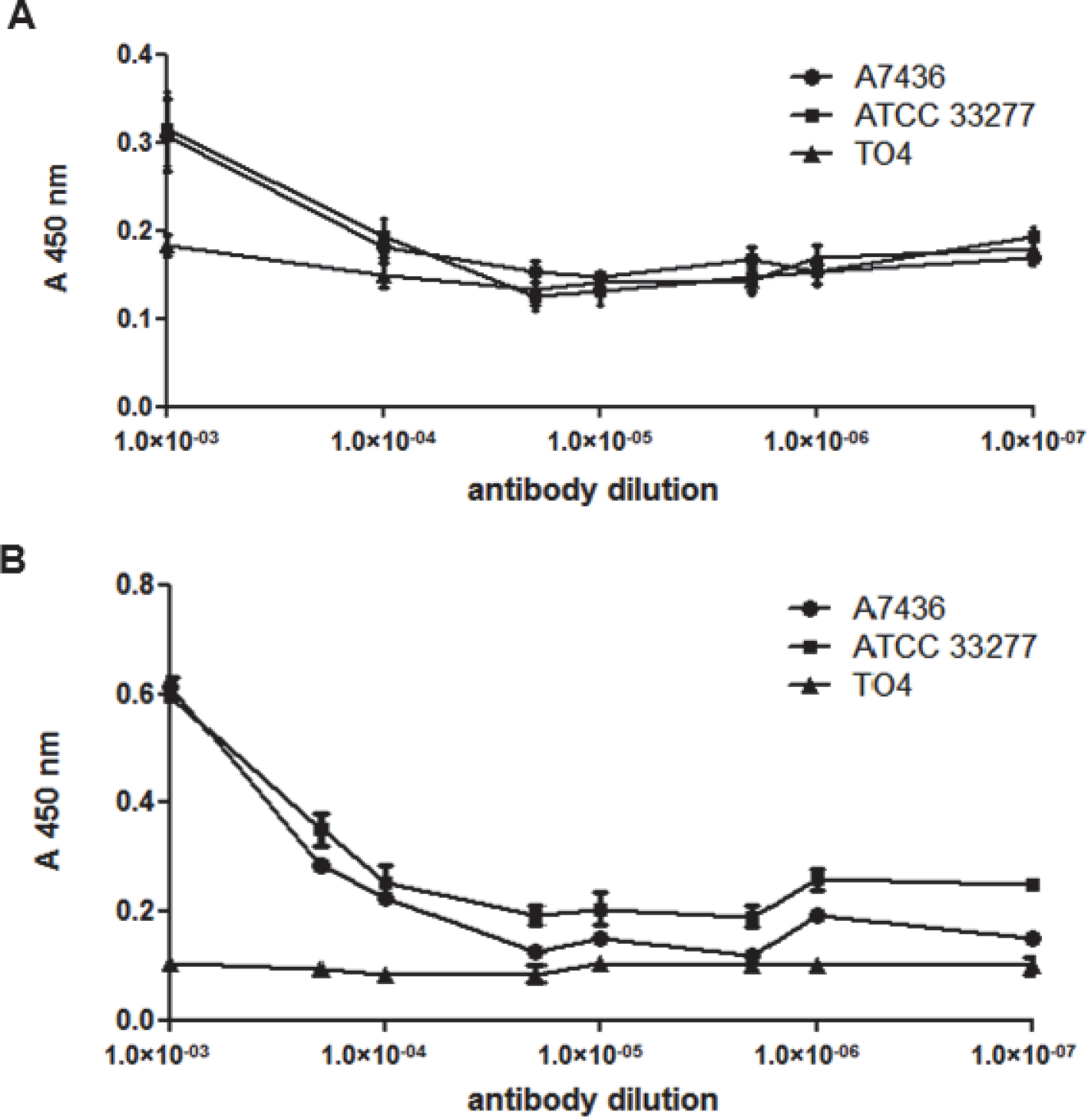
Reactivity of anti-HmuY antibodies with bacteria. ELISA was carried out using antibodies raised against purified HmuY protein (anti-HmuY 1) and *P*. *gingivalis* wild-type (A7436 and ATCC 33277) and *hmuY* mutant (TO4) strains. Bacteria were cultured in high iron/heme (A) or low-iron/heme (B) media. No reactivity was observed for anti-HmuY 1–1 (epitope 1), anti-HmuY 1–2 (epitope 2), or anti-HmuY 1–3 (epitope 3) antibodies. Live bacteria were used as antigens.

Characterization of antibodies was also carried out using native, non-denatured antigens. In agreement with Western blotting results, only antibodies raised against the purified HmuY protein or raised against epitope 2 reacted with HmuY protein used as an antigen, as determined by the ELISA assay (Fig. 7). When purified HmuY homologs from *P*. *intermedia* and *T*. *forsythia* were used as antigens, no reactivity with antibodies directed against purified HmuY protein (Fig. 8A) or epitope 2 (Fig. 8B) was observed. In addition, there was no reactivity when antibodies raised against epitope 1 and 3 were used (data not shown). When live *P*. *gingivalis* bacteria were used as antigens in the ELISA assay, only reactivity with antibodies raised against purified HmuY protein was visible (Fig. 9), especially in bacteria which were starved of iron and heme (Fig. 9B), compared with bacteria cultured under high-iron/heme conditions (Fig. 9A). As a negative control we used the *hmuY* mutant strain (TO4), which does not produce the HmuY protein. Importantly, no reactivity was found when live cells of *P*. *intermedia* or *T*. *forsythia* were used as antigens (data not shown).

In conclusion, the results obtained in this study demonstrate that *P*. *gingivalis* HmuY protein may serve as an antigen for specific determination of antibodies raised against this bacterium. Therefore, purified HmuY protein will be used to determine levels of anti-*P*. *gingivalis* antibodies in sera of patients with chronic periodontitis and patients with other forms of periodontal diseases. These studies are underway in our laboratory.
